# A Mechanistic Model of Cry2Ab12 Toxicity Against *Myzus persicae* via HSP60-Mediated OLA1 Inhibition

**DOI:** 10.3390/toxins18070279

**Published:** 2026-06-24

**Authors:** Xiaodi Zhao, Xuemei Hong, Liang Jin, Yi Lin

**Affiliations:** 1 Fujian Provincial Key Laboratory of Biochemical Technology, Department of Bioengineering & Biotechnology, College of Chemical Engineering, Huaqiao University, Xiamen 361021, China; 12231405@mail.sustech.edu.cn (X.Z.); hxmei@hqu.edu.cn (X.H.); 2 Fujian Key Laboratory of Ecology-Toxicological Effects & Control for Emerging Contaminants, Key Laboratory of Ecological Environment and Information Atlas, College of Environmental and Biological Engineering, Putian University, Putian 351100, China; 3 Henan Province Key Laboratory of Efficient Crop Production and Food Quality Safety, Zhoukou Normal University, Zhoukou 466001, China; 4 Modern Agricultural Industry Research Institute of Henan Zhoukou Agricultural High-Tech Industry Demonstration Zone, Dancheng County, Zhoukou 477150, China

**Keywords:** *Bacillus thuringiensis*, *Myzus persicae*, Obg-like ATPase 1 (OLA1), heat shock protein 60 (HSP60), mechanism

## Abstract

*Bacillus thuringiensis* Cry toxins are well known for their high insecticidal activity against Lepidoptera, Diptera, and Coleoptera and have been widely used in Bt transgenic crops. However, their activity against Hemipteran aphids remains relatively low. Identifying novel Cry proteins and elucidating their action mechanisms can facilitate the development of effective aphid control strategies. In this study, we found that ingestion of Cry2Ab12 did not kill *Myzus persicae* adults but significantly reduced their offspring number and exerted a lethal effect on *M. persicae* nymphs. After identifying Cry2Ab12 toxin-binding proteins in *M. persicae*, we further characterized the interaction with Obg-like ATPase 1 (OLA1), a conserved protein involved in growth regulation. Bio-layer interferometry (BLI), ELISA, and enzyme activity assays revealed that Cry2Ab12 and OLA1 do not interact directly. Interestingly, heat shock protein 60 (HSP60) was shown to mediate the interaction among Cry2Ab12, HSP60, and OLA1, leading to inhibition of OLA1 enzymatic activity. Based on these findings and bioinformatics simulations, we proposed a mechanistic model for Cry2Ab12 toxicity against *M. persicae*: upon ingestion of a sufficient amount of Cry2Ab12, the formation of the Cry2Ab12–HSP60–OLA1 complex impairs the cellular stress response, disrupts normal OLA1 expression, and ultimately restricts larval growth and development, resulting in lethality. This study provides new insights into the action of Cry toxins in aphids and offers a basis for developing enhanced aphid biocontrol strategies.

## 1. Introduction

*Bacillus thuringiensis* (Bt) insecticidal crystal proteins, especially Cry toxins, exhibit distinct activities against insects across various orders, mainly Lepidoptera, Diptera, Coleoptera, Hymenoptera, and Hemiptera. Additionally, some Cry toxins are active against nematodes, snails, and even cancer cells, while Cyt toxins—another group of delta-endotoxins alongside Cry toxins are primarily active against dipteran insects [1,2,3]. Owing to their unique insecticidal actions, Cyt and Cry toxins are widely used as insecticidal proteins for pest control globally. Whether applied as spray products or expressed in transgenic Bt crops, they effectively protect plants from insect damage and reduce the use of chemical insecticides [4,5]. Transgenic Bt crops, however, are not highly effective against hemipteran species like aphids [6,7]. Unlike lepidopteran, coleopteran, or dipteran pests, hemipterans possess piercing-sucking mouthparts, which fundamentally alter their interaction with Bt toxins [7]. This feeding mode limits their exposure to toxins expressed in transgenic plants or applied as sprays, as they primarily ingest phloem sap rather than whole plant tissues [7]. Consequently, both the practical efficacy and the evolutionary trajectory of Bt-based control strategies differ significantly for hemipterans compared to other pest orders [8,9]. Furthermore, studies have shown that insects can gradually evolve resistance to Bt toxins in controlled laboratory environments and Bt spray formulations in the field, indicating that the evolution of pest resistance is a important threat to the long-term and effective pest control by Bt crops [8,9,10,11]. Therefore, understanding how Bt toxins interact with insects and how insect defense systems respond to intoxication provides a foundation for better clarifying how insects become resistant to Bt toxins at the mechanistic level.

Cry proteins have achieved certain success in controlling insects such as Lepidoptera, Coleoptera, and Diptera, but their efficacy against hemipteran insects has been consistently poor. Insect intestinal pH and protease environment are crucial factors for Cry protein activation [12]. Unlike lepidopteran insects, aphids have an acidic intestinal pH, which hinders the first step of Cry protein toxicity [13,14]. To address this phenomenon, a study fed pre-activated Cry proteins to aphids, which successfully bound to the brush border membrane vesicles (BBMVs) of the aphid midgut; however, no aphicidal activity was detected [15]. Currently, only Cry1Cb2 and Cry41-related proteins are known to exhibit good insecticidal activity against *Myzus persicae* [16,17]. Among them, Cry41-related proteins may exert aphicidal effects by binding to cathepsin B in *M. persicae* and enhancing its enzymatic activity [18].

Obg-like ATPase 1 (OLA1) is a P-loop GTPase belonging to the Obg subfamily, YchF subgroup, and the translation factor—related (TRAFAC) class, possessing both GTPase and ATPase activities [19,20]. Accumulating evidence indicates that OLA1 is a central regulator of cellular stress responses, encompassing multiple adaptive mechanisms that are critical for maintaining protein homeostasis during stress. In mammals as well as in *Saccharomyces cerevisiae*, it participates in the translational control of cell proliferation, growth, and protein synthesis [21,22]. Marta et al. found that after knockout of the YchF homologous protein in yeast, most results showed non-lethal changes in the knockout yeast [23]. Monika et al. reported similar progress: RNAi-mediated knockdown of the TcYchF gene in trypanosomes inhibited its protein expression, leading to significant suppression of trypanosome growth [24]. Additionally, studies have performed RNAi—mediated knockdown of OLA1 protein expression in various human—derived cell lines, yielding inconsistent results—some cell lines showed no effect on proliferative capacity, while others exhibited growth inhibition [19,25,26]. Both *ola1* gene knockout mice and *ola1* gene knockout mouse embryos exhibit cell cycle arrest and growth restriction. This arrest reduces cell proliferation by regulating the translation of p53 and p21, leading to impaired embryonic growth and development, subsequent perinatal death of mice, and overall growth retardation and developmental delay [22,26]. OLA1, a cytoplasmic protein, plays a regulatory role in the secretion pathways of multiple proteins. It has also been identified as a functional protein involved in regulating cell–matrix adhesion, which can induce significant changes in cell adhesion and related phenotypes [20]. A study revealed that OLA1 is implicated in modulating the proliferation and apoptosis of cells in hepatocellular carcinoma—reduced OLA1 expression significantly inhibits the proliferation, migration, invasion, and tumorigenicity of hepatocellular carcinoma cells [27]. Protein—protein interaction analysis showed that OLA1 can bind to the variable C-terminal domain of HSP70, hindering the interaction between HSP70 and the E3 ubiquitin ligase CHIP, this prevents CHIP—mediated ubiquitination of HSP70, thereby stabilizing HSP70 and protecting cells from heat shock [28]. When OLA1 expression is low in cells, the affinity between the HSP70—SOD2 complex and ubiquitin ligase III is enhanced, promoting the degradation of SOD2 and creating an oxidative stress environment. Under high temperature conditions, overexpression of OLA1 enhances the binding ability between SOD2 and HSP70, indicating that OLA1 facilitates the formation of the SOD2—HSP70 complex [29]. In yeast, YBR025c/OLA1 is regarded as a proteasome—associated protein capable of interacting with the 26S proteasome [30,31]. However, this function of OLA1 has not been observed in human cells [32].

Despite these advances, a significant knowledge gap remains: the molecular mechanisms by which Cry toxins interact with cellular targets in hemipterans, particularly aphids, are largely unknown. Moreover, while OLA1 has been implicated in stress responses and protein homeostasis in model organisms, its potential role as a Cry toxin-binding protein and its contribution to aphid physiology during toxin exposure have never been explored. Given the unique feeding mode of hemipterans and their poor susceptibility to most Cry toxins, we hypothesized that Cry toxins may exhibit differential activity against different developmental stages of *M. persicae*, and OLA1 could serve as a putative binding partner for Cry2Ab12 in aphids, potentially modulating toxin effects through interactions with other cellular proteins such as HSP60. To test these hypotheses, this study aimed to: (1) Evaluate the insecticidal activity of the Cry2Ab12 toxin against *M. persicae* adults and nymphs; (2) Identify and validate OLA1 as a Cry2Ab12-binding protein from aphid homogenates; (3) Investigate the effect of Cry2Ab12 on OLA1 enzymatic activity, alone and in combination with HSP60. Collectively, these findings provide new insights into the molecular basis underlying the relative insensitivity of aphids to Cry toxins and identify OLA1 as a potential target for future pest control strategies.

## 2. Results

### 2.1. Expression, Purification and Toxicological Characterization of Recombinant Cry2Ab12 Toxin

**Figure 1:**
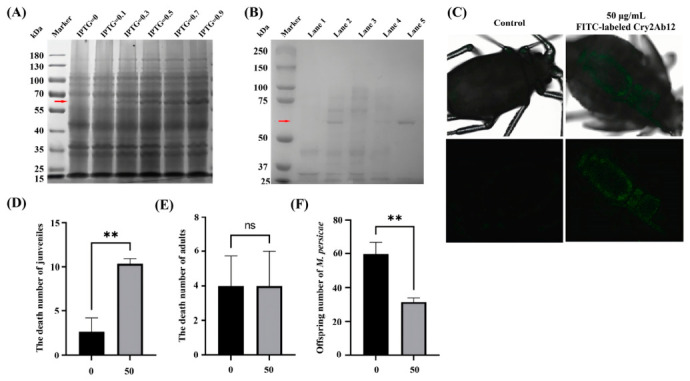
Expression, purification and toxicological characterization of recombinant Cry2Ab12 toxin. (**A**) SDS-PAGE analysis of Cry2Ab12 toxin expression in *E. coli*. Total bacterial lysates collected after protein induction with a range of IPTG concentrations are presented. (**B**) Purification of Cry2Ab12 toxin. Lane 1: denatured inclusion bodies without protein induction by IPTG; lane 2: denatured inclusion bodies after protein induction by 0.9 mmol/L IPTG at 25 °C; lane 3: Denatured inclusion effluent from the column; lane 4: fraction from washing with 20 mmol/L imidazole; lane 5: purified Cry2Ab12 toxin eluted with 200 mmol/L imidazole. Arrows point to the position of the Cry2Ab12 target protein bands. (**C**) Distribution in vivo of different concentrations of FITC-labeled Cry2Ab12 protein ingested by *M. persicae* at 40 min. (**D**) Insecticidal effects of different concentrations of Cry2Ab12 toxin on *M. persicae* juveniles. (**E**) Insecticidal effects of different concentrations of Cry2Ab12 toxin on *M. persicae* adults. (**F**) Lethal effects of Cry2Ab12 toxin across a range of concentrations on the F1 generation of treated groups. A statistically significant difference between the two groups was identified using Student’s *t*-test, as indicated by **(*p* < 0.01).

The pET-30a(+) expression vector was used to clone the cDNA encoding the Cry2Ab12 toxin, enabling recombinant protein production in *E. coli*. Sodium dodecyl sulfate–polyacrylamide gel electrophoresis (SDS-PAGE) analysis confirmed that the expression of Cry2Ab12 toxin was induced by 0.9 mmol/L IPTG at 25 °C; most of the toxin was recovered from the insoluble fraction, whereas a small amount was detected in the soluble fraction (Figure 1A,B). Inclusion bodies harboring Cry2Ab12 toxin were dissolved in denaturing buffer and passed through a 0.45 µm membrane filter. The denatured toxin was subsequently purified using Ni-NTA resins and subjected to refolding. SDS-PAGE was employed to analyze the purified Cry2Ab12 toxin (Figure 1B).

Subsequently, FITC-labeled Cry2Ab12 toxin was used for detection and visualization of distribution of Cry2Ab12 toxins in vivo by Confocal Microscopy. The aphids fed on the artificial diet containing 0 μg/mL FITC-labeled Cry2Ab12 toxin did not produce fluorescence in vivo, while green fluorescence appeared in the middle of the body of the aphid fed on the artificial diet containing 50 μg/mL FITC-labeled Cry2Ab12 toxin (Figure 1C). These results confirmed that Cry2Ab12 toxin could successfully enter the aphid by the feeding method in the study. Then, the use of a small bag of parafilm membranes containing the artificial diet for aphids and Cry2Ab12 toxin in different concentrations were designed to rear *M. persicae*, and thus bioassays of Cry2Ab12 toxin were performed against adults and nymphs (first instar nymphs) of *M. persicae*, respectively. The striking differences in mortality rates of aphids on the artificial diet with 50 μg/mL Cry2Ab12 toxin were found between adults and nymphs (Figure 1D,E), suggesting that the Cry2Ab12 toxin had a significant lethal effect on *M. persicae* nymphs but showed the opposite effect on adults. Interestingly, offspring numbers of *M. persicae* adults treated with Cry2Ab12 toxin were significantly decreased in comparison with the control group (Figure 1F), indicating that Cry2Ab12 toxin could affect the reproduction of *M. persicae* adults.

### 2.2. Identification of Cry2Ab12 Toxin-Binding Proteins in M. persicae

**Figure 2:**
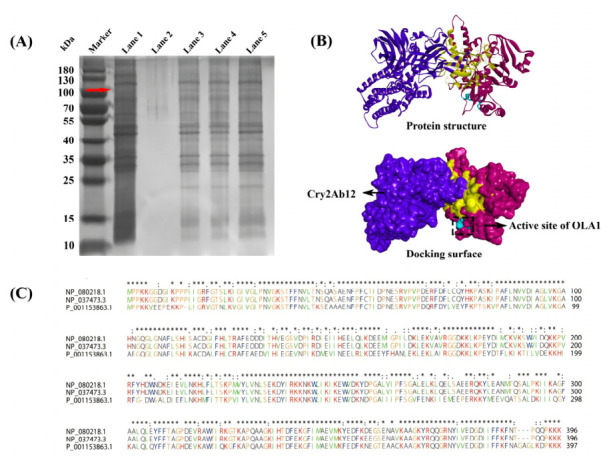
Identification of OLA1 as a Cry2Ab12 toxin-binding protein from *M. persicae* homogenates. (**A**) Pull-down assay. M: Protein ladder; Lane 1: CNBr-activated Sepharose^TM^ 4B + *M. persicae* homogenates (negative control); Lane 2: CNBr-activated Sepharose^TM^ 4B + PBS (blank control); Lanes 3–5: Cry2Ab12 toxin-CNBr-activated Sepharose^TM^ 4B + *M. persicae* homogenates (three independent pull-down replicates). Arrow indicates the target band of Cry2Ab12 toxin-binding proteins. (**B**) Molecular homology and multiple-sequence alignment of OLA1 from different species. NP-080218.1 (*Mus musculus*); NP-037473.3 (*Homo sapiens*); P-001153863.1 (*Myzus persicae*, identified by database matching to *Acyrthosiphon pisum*); Asterisks (*) denote conserved residues. (**C**) Molecular docking structure and binding interface of Cry2Ab12 toxin (purple) with OLA1 (red). The binding interface is highlighted in yellow. Highly conserved residues within the active site of OLA1 (Asn-31, Gly-33, Lys-34, Ser-35, Thr-36, Asn-228, Leu-229, Glu-231, Ser-262, Gly-263, and Val-264) are shown in blue.

To isolate Cry2Ab12 toxin-binding proteins from *M. persicae* homogenates, the purified Cry2Ab12 toxin was used to generate toxin-coupled Sepharose™ 4B beads. In comparison with control beads, the Cry2Ab12 toxin effectively pulled down protein bands at approximately 100 kDa (Figure 2A). Then, the Cry2Ab12 toxin-binding proteins were identified as tubulin beta chain, elongation factor 1-gamma, OLA1, elongation factor 1-alpha, 26S protease regulatory and H/ACA ribonucleoprotein (Table 1). Among all the proteins identified, OLA1 was remarkable for its relatively high MASCOT score that bound to Cry2Ab12 toxin. OLA1 is a central regulator of cellular stress response, which encompasses a variety of adaptive mechanisms that are essential for maintaining protein balance during stress, and participate in the regulation, at the translation level, of cellular proliferation, expansion, and polypeptide biosynthesis in mammals and *S. cerevisiae* [21,22]. It is also reported that losing *ola1* gene had a great impact on development and survival of mouse embryos [22,23]. Sequence comparison was performed for homologous analysis of three different sources of OLA1 protein from *Mus musculus*, *Homo sapiens*, and *Acyrthosiphon pisum*. Multiple sequence alignment of OLA1 from aphid and other species confirmed that OLA1 was a highly conserved protease (Figure 2B), which is consistent with literature reports showing highly conserved gene and protein sequences of OLA1 in yeast, mice, and humans [33]. Therefore, the reported functions of mouse OLA1 protein could serve as a reference for understanding the OLA1 function in *M. persicae*.

In addition, the quality of the Cry2Ab12 and OLA1 protein models was validated using MolProbity, yielding clashscores of 4.73 (94th percentile) and 4.99 (94th percentile), respectively. Molecular docking analysis was undertaken subsequently to resolve the details of the interaction between Cry2Ab12 toxin and OLA1 (Figure 2C). In the activated Cry toxin, domain II is composed of a β-prism structure with three antiparallel β-sheets surrounding a hydrophobic core, a configuration critical for receptor binding [34]. In Figure 2C, OLA1 interacted with domain II of Cry2Ab12, and the G1-5 box region or the active site of OLA1 (blue region: Asn-31, Gly-33, Lys-34, Ser-35, Thr-36, Asn-228, Leu-229, Glu-231, Ser-262, Gly-263, Val-264) showed no interaction with Cry2Ab12 toxin. The binding interface between Cry2Ab12 and OLA1 (yellow region in Figure 2C) is localized to Switch I region, Switch II region, and Ych-GTPase_C domain of OLA1. Sequence alignment across three OLA1 orthologs confirmed that Cry2Ab12-binding residues in the Switch I region are evolutionarily conserved, while only Lys-114 in the Switch II region exhibits variability (Figure 2B), indicating that Cry2Ab12 targets a conserved domain of OLA1. Previous study reported that Switch I region and Switch II region mediate interactions with the γ-phosphate group of bound GTP, inducing conformational changes essential for efficient GTP hydrolysis [35]. Binding of Cry2Ab12 to OLA1 encompasses both the Switch I region and Switch II region; therefore, it is likely that Cry2Ab12 could modulate GTP hydrolysis kinetics of OLA1 without abolishing its catalytic activity.

### 2.3. Transcriptional Dynamics of M. persicae OLA1 Across Developmental Stages and in Response to Cry2Ab12 Toxin Exposure

**Figure 3:**
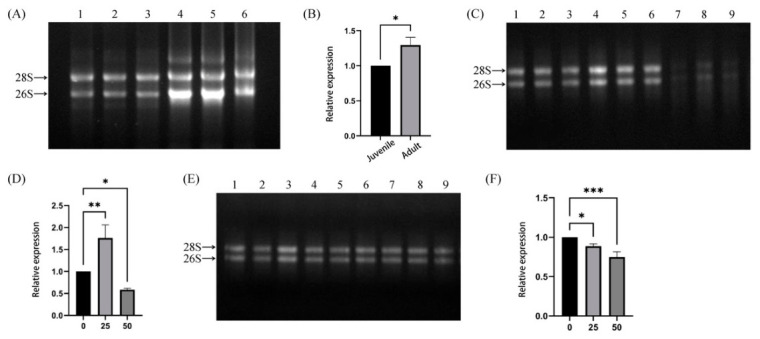
Transcriptional dynamics of OLA1 in *M. persicae* across developmental stages and in response to Cry2Ab12 toxin exposure. (**A**) Total RNA electrophoresis of adult and juvenile *M. persicae*. Lanes 1–3: adult samples; lanes 4–6: juvenile samples. (**B**) Relative OLA1 transcript levels in adult versus juvenile *M. persicae*. The transcript level in juveniles was set to 1, and that in adults was 1.3-fold higher. (**C**) Total RNA electrophoresis of juvenile *M. persicae* following Cry2Ab12 toxin treatment. Lanes 1–3: 0 μg/mL; lanes 4–6: 25 μg/mL; lanes 7–9: 50 μg/mL. (**D**) Effect of Cry2Ab12 toxin on OLA1 transcript levels in juvenile *M. persicae*. The transcript level in the 0 μg/mL treatment group was set to 1. Relative transcript levels were approximately 1.8-fold at 25 μg/mL and 0.6-fold at 50 μg/mL. (**E**) Total RNA electrophoresis of adult *M. persicae* following Cry2Ab12 toxin treatment. Lanes 1–3: 0 μg/mL; lanes 4–6: 25 μg/mL; lanes 7–9: 50 μg/mL. (**F**) Effect of Cry2Ab12 toxin on OLA1 transcript levels in adult *M. persicae*. The transcript level in the 0 μg/mL treatment group was set to 1. Relative transcript levels decreased by 11% at 25 μg/mL and by 25% at 50 μg/mL (***, *p* < 0.001; **, *p* < 0.01; *, *p* < 0.05).

To study the function of the *ola1* gene in *M. persicae*, we sequenced the transcriptomes of juvenile and adult aphids to compare *ola1* expression patterns. RT-qPCR was performed to quantify *ola1* transcript levels in normally developing juveniles and adults, with total RNA quality verified by electrophoresis (Figure 3A). Interestingly, the transcriptional level in adults was approximately 1.3-fold higher than in juveniles (Figure 3B). This stage-specific difference may underlie the distinct sensitivities of juveniles and adults to Cry2Ab12 toxin, suggesting that at the same toxin concentration, juveniles experience a stronger biological effect due to their lower basal *ola1* expression. Indeed, bioassays confirmed that Cry2Ab12 toxin exhibited specific insecticidal activity against juveniles but not adults (Figure 1D,E).

Juvenile aphids were then fed with Cry2Ab12 toxin at 0 (control), 25, or 50 μg/mL, and *ola1* transcript levels were quantified after 24 h. The results revealed a biphasic response (Figure 3C,D). Exposure to 50 μg/mL Cry2Ab12 toxin significantly reduced *ola1* transcription in juveniles, which likely contributes to growth retardation and subsequent lethality. Conversely, exposure to 25 μg/mL Cry2Ab12 toxin led to an upregulation of *ola1* transcription, a phenomenon possibly linked to hormesis—defined as a biphasic dose–response curve featuring low-dose stimulation and high-dose inhibition [36]. Compared to the control group, 25 μg/mL Cry2Ab12 toxin increased *ola1* transcription by 76%, whereas 50 μg/mL reduced it by 42% (Figure 3D). We hypothesized that at low toxin concentrations, juveniles mount a defensive response without inducing oxidative stress. Although Cry2Ab12 binding compromises OLA1 enzymatic activity, juveniles upregulate *ola1* transcription to compensate for the reduced catalytic function, thereby maintaining overall OLA1 activity. Previous study demonstrated that such OLA1 overexpression augments cellular tolerance to heat shock [28]. Similarly, adult aphids were fed with 0, 25, or 50 μg/mL Cry2Ab12 toxin, and *ola1* transcript levels were quantified after 24 h. Electrophoresis confirmed total RNA quality, revealing a dose-dependent decrease in *ola1* expression (Figure 3E,F). Adult aphids possess fully developed tissues and require minimal cell proliferation, which may reduce their dependence on *ola1*—mediated mitotic regulation. While *ola1* downregulation in adults does not affect meiosis, it may disrupt mitosis-related embryonic development, directly explaining why offspring of Cry2Ab12—exposed adults exhibit growth inhibition and lethality (Figure 1F).

### 2.4. Lack of Direct Interaction Between Cry2Ab12 and OLA1 In Vitro

**Figure 4:**
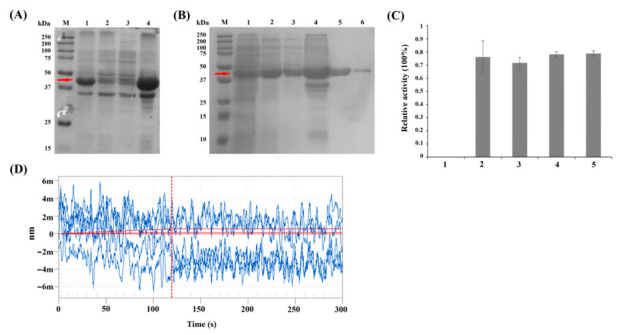
Expression, purification, and interaction of recombinant OLA1 with the Cry2Ab12 toxin. (**A**) Expression of *M. persicae* OLA1 in *E. coli*. Lane 1: total bacterial lysate after induction with 0.5 mmol/L IPTG; lane 2: total bacterial lysate without IPTG induction (0 mmol/L); lane 3: pellet containing OLA1 inclusion bodies; lane 4: supernatant of bacterial lysate. (**B**) Purification of *M. persicae* OLA1. Lane 1: supernatant containing soluble OLA1; lanes 2–3: purified soluble OLA1 eluted with 200 mmol/L imidazole; lane 4: denatured inclusion bodies containing OLA1; lanes 5–6: purified OLA1 from the 200 mmol/L imidazole elution fraction. (**C**) Effect of Cry2Ab12 toxin on OLA1 enzymatic activity with GTP substrate. Group 1 (blank control): 50 μL buffer only; Group 2: 40 μL buffer + 5 μL OLA1 + 5 μL substrate; Group 3: 35 μL buffer + 5 μL Cry2Ab12 + 5 μL OLA1 + 5 μL substrate; Group 4: 30 μL buffer + 10 μL Cry2Ab12 + 5 μL OLA1 + 5 μL substrate; Group 5: 25 μL buffer + 15 μL Cry2Ab12 + 5 μL OLA1 + 5 μL substrate. (**D**) Binding kinetics of Cry2Ab12 toxin to OLA1 measured by the Octet system. Cry2Ab12 concentrations ranged from 62.5 to 1000 nmol/L.

The complementary DNA (cDNA) for *M. persicae* OLA1 was synthesized with codon optimization tailored for heterologous expression in *E. coli*. Following induction by 0.5 mmol/L IPTG, SDS-PAGE analysis revealed that recombinant OLA1 was expressed after 16 h and partitioned into both the soluble fraction and inclusion bodies (Figure 4A). Filtration of the soluble fraction was performed through a 0.45 µm filter, while the inclusion bodies were first solubilized in denaturing buffer and then similarly filtered. Both fractions were purified separately using Ni-NTA resins. Subsequently, refolding of the denatured OLA1 protein was carried out in 20 mmol/L Tris-HCl supplemented with 2 mmol/L reduced glutathione and 0.2 mmol/L oxidized glutathione, across a range of pH values (8.0, 7.5, 7.2, 7.0) and urea concentrations (6, 4, 2, 0 mol/L). Homogeneous purification of recombinant OLA1 was achieved, as demonstrated by SDS-PAGE analysis (Figure 4B). The soluble fraction yielded more heterogeneous proteins than the denatured inclusion bodies; therefore, the refolded inclusion body protein was used in subsequent experiments.

Qualitative and quantitative measurements of the Cry2Ab12 toxin-OLA1 interaction were carried out in vitro. Using GTP as the substrate, the effect of Cry2Ab12 on OLA1 enzymatic activity was examined. As shown in Figure 4C, different concentrations of Cry2Ab12 had almost no noticeable effect on OLA1 activity, indicating no statistically significant positive correlation between Cry2Ab12 concentration and the relative enzymatic activity of OLA1. Thus, the addition of Cry2Ab12 toxin alone does not significantly affect OLA1 activity. Quantitative analysis of the interaction between OLA1 and Cry2Ab12 (at concentrations increasing from 62.5 to 1000 nmol/L) was then carried out using ForteBio bio-layer interferometry (Figure 4D and Table 2). These results were in line with the quantitative binding data in Figure 4C: as the concentration of OLA1 increased, the binding signal in Figure 4D showed no statistically significant positive correlation, confirming the absence of a direct interaction between Cry2Ab12 and OLA1 in vitro. Considering that OLA1 was found to bind Cry2Ab12 in pull-down experiments conducted with *M. persicae* homogenates, we speculated that the interaction between Cry2Ab12 and OLA1 may occur in the form of a protein complex in vivo, which would explain why the two proteins do not interact directly when expressed individually in vitro.

### 2.5. HSP60 Mediated the Interaction Between Cry2Ab12 and OLA1

**Figure 5:**
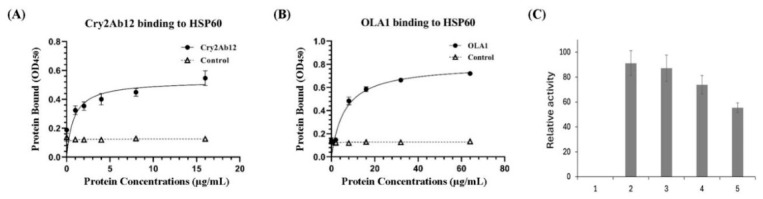
HSP60 mediates the interaction between Cry2Ab12 and OLA1. (**A**,**B**) ELISA analysis of binding interactions. (**A**) Binding of biotinylated Cry2Ab12 to immobilized HSP60. (**B**) Binding of biotinylated OLA1 to immobilized HSP60 [38]. Nonspecific binding controls were performed in parallel. Each experiment was performed three times. (**C**) Effect of the Cry2Ab12-HSP60 complex on OLA1 enzymatic activity with GTP substrate. Group 1 (blank control): 40 μL buffer + 10 μL substrate; Group 2: 30 μL buffer + 10 μL OLA1 + 10 μL substrate; Group 3: 20 μL buffer + 10 μL Cry2Ab12 + 10 μL OLA1 + 10 μL substrate; Group 4: 20 μL buffer + 10 μL HSP60 + 10 μL OLA1 + 10 μL substrate; Group 5: 10 μL buffer + 10 μL Cry2Ab12 + 10 μL HSP60 + 10 μL OLA1 + 10 μL substrate.

HSP60 has been reported to participate in the early embryogenesis of *Drosophila* (Diptera: Drosophilidae) and may act as a chaperone in interactions with OLA1, thereby helping to maintain OLA1 function and normal development of *M. persicae* [37,38]. The binding interactions among Cry2Ab12, HSP60, and OLA1 were determined by ELISAs. With increasing concentrations of biotin-labeled Cry2Ab12, more protein bound to immobilized HSP60, and binding reached saturation at 16 μg/mL Cry2Ab12 (Figure 5A). Similarly, increasing concentrations of biotin-labeled OLA1 bound to immobilized HSP60, with saturation observed at 64 μg/mL (Figure 5B) [38]. These results suggested that although OLA1 was found to bind Cry2Ab12 in pull-down experiments, its enzymatic activity is not directly affected by Cry2Ab12 alone—likely because HSP60 is required as an intermediate mediator to form a ternary complex. To investigate this, OLA1 was incubated together with GTP and the reaction cocktail, and the liberation of inorganic phosphate resulting from GTP hydrolysis was tracked by recording the rise in absorbance at 620 nm. Figure 5C indicated that the enzymatic function of OLA1 remained unchanged upon addition of Cry2Ab12 alone, which aligned with the findings presented in Figure 4C. In contrast, OLA1 activity was slightly reduced by the addition of HSP60 alone and significantly downregulated when both Cry2Ab12 and HSP60 were added simultaneously. Based on these findings, we hypothesize that Cry2Ab12 does not directly bind to or act on OLA1. Instead, it likely forms a ternary complex by first binding to HSP60 and subsequently recruiting OLA1. This complex functions as the active unit, ultimately mediating the unique aphid-suppressive activity of Cry2Ab12 demonstrated in previous experiments.

## 3. Discussion

It is well established that *B. thuringiensis* Cry toxins exhibit potent insecticidal activity toward insects of the orders Lepidoptera, Coleoptera, and Diptera but exhibit low activity against Aphididae [1,2,39]. Recent studies have elucidated the physiological basis for the innate low susceptibility of aphids to most Cry toxins: the digestive tract of *A. pisum* harbors highly efficient cysteine proteases that rapidly degrade Cry toxins [15]. The Cry41-related toxin has been shown to act as an allosteric activator of cathepsin B, thereby exerting insecticidal activity against aphids [18]. In *M. persicae*, resistance to Cry7Ab4 toxin involves HSP60-mediated cellular defense pathways, in which OLA1 plays a key regulatory role [38]. Given the ongoing global effort to discover novel Cry toxins, understanding their mechanisms of action is essential, particularly in the context of increasing insect resistance. To date, how Cry2Ab functions and how insects develop resistance to it remain unclear. In this study, we uncovered the unique aphid-suppressive characteristics of the Cry2Ab12 toxin. Toxicity bioassays revealed that the active Cry2Ab12 toxin had no lethal effect on *M. persicae* adults but did exhibit moderate inhibitory effects on the survival of nymphs and on the survival of offspring from toxin-fed adults. This differential susceptibility was likely interpreted considering the piercing-sucking feeding behavior of aphids. Unlike chewing insects that ingest large amounts of leaf tissue, aphids feed continuously on phloem sap, leading to low-volume toxin intake. Nymphs, with their smaller size and less developed stress responses, are more vulnerable to this chronic exposure [15,16,17]. This feeding mode directly limits field effectiveness, as even active Cry toxins may not reach lethal concentrations in phloem sap under real agricultural conditions [12,17].

We then identified several Cry2Ab12 toxin-binding proteins in *M. persicae*, including OLA1, 26S protease regulatory subunit, and H/ACA ribonucleoprotein. Among these, we selected OLA1 for further study because it is a highly conserved protein across species, performs similar functions in different organisms, and is strongly associated with growth and developmental regulation in previous reports [25,26,40]. Molecular docking simulations revealed that Cry2Ab12 bound to the Switch I and Switch II regions of OLA1 but does not overlap with its enzymatic active site. This finding is consistent with subsequent enzymatic activity assays, which showed that the addition of Cry2Ab12 did not affect OLA1 enzymatic activity. Furthermore, quantitative interaction analysis confirmed that Cry2Ab12 and OLA1 do not directly interact. Given that prior studies have explored the functional modality of OLA1 in protein complex-mediated actions [28,38], we hypothesized that the growth and developmental inhibition of *M. persicae* nymphs by Cry2Ab12 toxin is associated with OLA1 but occurs through indirect mechanisms rather than direct interaction. Specifically, this process likely involves the assembly of a multi-protein complex through which Cry2Ab12 exerts its effects via complex-mediated signaling.

Previous studies have shown that OLA1 interacts with the variable C-terminal region of HSP70, hindering the binding of CHIP—an E3 ligase that targets HSP70. By preventing CHIP-dependent ubiquitination, OLA1 stabilizes HSP70 and increases its expression levels, thereby enhancing cellular viability during heat stress [29]. HSP60 has been identified as a Cry7Ab4-binding protein using pull-down assays and plays a specific role in insect defense against Cry toxins [38]. Compared to HSP70, the function of HSP60 in insects has been far less extensively studied, especially regarding its interactions with Bt toxins. In this study, we found that the addition of HSP60 alone exerted an inhibitory effect on the enzymatic activity of OLA1. Moreover, the co-addition of HSP60 and Cry2Ab12 resulted in a more pronounced inhibitory effect than that of HSP60 alone. This result supported our hypothesis that the growth and developmental inhibition of *M. persicae* nymphs by Cry2Ab12 toxin involved the binding of multiple proteins and was mediated through the formation of a protein complex.

The Cry2Ab12 toxin-binding proteins identified in *M. persicae* include OLA1, H/ACA ribonucleoprotein, and the 26S protease regulatory subunit. OLA1 is a cytoplasmic protein that regulates the secretion of various proteins; it controls cell–matrix adhesion, CDK2 expression, and SOD2 activity, and mediates the ubiquitination and degradation of HSP70 [27,28,29]. H/ACA ribonucleoprotein primarily directs site-specific pseudouridylation of target RNAs and is crucial for pre-mRNA splicing, ribosome biogenesis, and telomere maintenance [41]. The 26S protease plays a role in cell cycle regulation and is indispensable for cell growth; additionally, it is also a ubiquitin-dependent major pathway for protein degradation [42,43].

**Figure 6:**
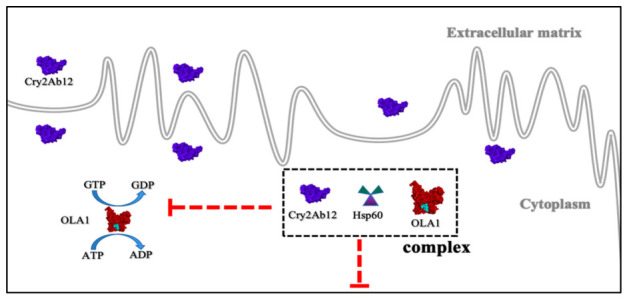
Proposed model for the inhibition of *Myzus persicae* nymphs growth and development by the Cry2Ab12 toxin. Upon entering the cell, the Cry2Ab12 toxin binds to HSP60 and OLA1, forming a ternary complex. This complex subsequently affects the ubiquitin-proteasome degradation pathway and impairs the intrinsic enzymatic activity of OLA1. The disruption of normal cellular stress responses, resulting from the formation of the Cry2Ab12-HSP60-OLA1 complex, alters the physiological expression level of OLA1 and ultimately restricts larval growth and development.

From the above outcomes, we proposed a hypothetical model for the aphid-inhibitory mechanism of the Cry2Ab12 toxin (Figure 6). Upon entering the cell, Cry2Ab12 binds to HSP60 and then interacts with OLA1 through HSP60, forming a complex-like structure that acts on the cell. This structure impairs the ubiquitin-proteasome degradation pathway and reduces the enzymatic activity of OLA1. In adult *M. persicae*, the innate immune defense system is relatively well-developed. Adults exhibit rapid self-regulation of OLA1 expression upon exposure to stress, thereby preventing rapid lethality even after ingesting a certain amount of Cry2Ab12 toxin. In contrast, *M. persicae* nymphs are more dependent on the normal function of OLA1 than adults. After ingesting the toxin, the formation of the Cry2Ab12-HSP60-OLA1 complex impairs the stress response in nymphs, disrupts the normal expression level of OLA1, and ultimately restricts growth and development, leading to death.

## 4. Conclusions

This study revealed the distinct effects of the Cry2Ab12 toxin on different developmental stages of *M. persicae*, elucidated the critical role of OLA1 in this process, and proposed a unique mechanistic model for the specific anti-aphid characteristics of Cry2Ab12 toxin. These findings offered novel evidence and conceptual perspectives for elucidating the resistance mechanisms of aphids to Cry toxins. Regarding field usefulness, Cry2Ab12 exhibited sublethal, nymph-specific effects, making it suitable for integrated pest management (IPM) rather than standalone use. To implement biocontrol methods, field application should target peak nymph emergence, and delivery systems that enhance phloem exposure could improve efficacy. Additionally, OLA1 and HSP60 may serve as molecular targets for developing synergists to boost Cry toxicity. Future studies addressing the following question would be of great interest: after adult *M. persicae* ingest Cry2Ab12, tracking whether the toxin is present in their embryos and offspring nymphs could further validate the reproductive inhibition of the toxin on adults and refine the proposed mechanistic hypothesis.

## 5. Materials and Methods

Bacterial strains and insects. For molecular cloning and recombinant protein expression, *E. coli* strains DH5α and BL21 (DE3) (TransGen, Beijing, China) were employed, respectively. The same supplier’s ProteinIso Ni-NTA resins (TransGen, Beijing, China) were utilized to purify the resulting recombinant proteins. *M. persicae* aphids, initially collected in 2018 from a field in Fujian Province, China, were reared in our laboratory on *Brassica chinensis* L. at 23 ± 2 °C, 70–80% humidity, and under a 16:8 photoperiod.

Expression and purification of Cry2Ab12 toxin and OLA1. For amplification of the complete *cry*2Ab12 coding sequence (Accession No. ACC86136.1), primers were designed targeting the upstream region of the initiation codon (ATG) and the downstream region of the termination codon (TAC). The full-length cDNA encoding the Cry2Ab12 toxin was then amplified by PCR with primer pair F1 (5′-GGA TCC GAT GAA TAG CGT TC TTA ACA GC-3′)/R1 (5′-GTC GAC GTA CAG CGG GCT GAT GTT G-3′). This PCR product was subsequently ligated into the pET-32b(+) expression vector using *Noc* I and *Xho* I restriction sites, resulting in the recombinant plasmid designated pET-*cry*2Ab12. *Ola1* cDNA (Accession No. NP_001153863.1) was amplified by PCR with F2 primer (5′-GAT ATA CAT ATG CAC CAC CAT CA-3′) and R2 primer (5′-GGC CGC AAG CTT TCA TTA TT-3′) and subsequently inserted into the pET-30a(+) expression vector via the *Nde* I and *Hind* III restriction sites, generating the recombinant plasmid pET-*ola1*. After transforming the two recombinant plasmids individually into competent BL21(DE3) cells, positive clones were selected by colony PCR and grown at 37 °C in 100 mL LB medium containing 50 μg/mL kanamycin for both constructs. Induction of Cry2Ab12 and OLA1 expression was achieved by adding IPTG to final concentrations of 0.9 mmol/L and 0.5 mmol/L, respectively, upon reaching an OD_600_ of 0.8. The cultures were subsequently incubated at 25 °C with orbital shaking (180 rpm) for 16 h, followed by cell harvest via centrifugation (4000 rpm, 4 °C, 20 min) and disruption in 20 mmol/L Tris-HCl (pH 8.0) by sonication (3 s pulses on, 6 s off, for 20 min). Centrifugation of bacterial lysates (12,000 rpm, 4 °C, 10 min) was performed, followed by removal of the supernatant and collection of the inclusion bodies from the pellets. The inclusion bodies carrying His-tagged Cry2Ab12 toxin and OLA1 were denatured for 12 h in denaturing buffer (8 mol/L urea, 20 mmol/L Tris-HCl, pH 8.0) and then clarified through a 0.45 μm filter. Purification of the denatured Cry2Ab12 toxin and OLA1 was achieved using Ni-NTA resins by affinity chromatography according to the protocol of Yang et al. [44], with subsequent refolding performed as described by Marina et al. [45].

Localization of FITC-labeled Cry2Ab12 toxin in *M. persicae*. The active domain of the Cry2Ab12 toxin was expressed, purified, and quantified. The purified protein was transferred to brown light-protected glass vials, and FITC (Sigma-Aldrich, St. Louis, MO, USA) was added at a protein-to-dye mass ratio of 1:2. The mixture was stirred overnight (12–16 h) on ice. The overnight-incubated protein–dye mixture was placed into tinfoil-wrapped 100 mL centrifuge tubes, balanced, and centrifuged (6000 rpm, 4 °C, 15 min). The supernatant was aspirated into a dialysis bag, which was then immersed in a beaker containing 1 L of pre-chilled PBS buffer with a stir bar. The beaker was placed on an ice bath atop a magnetic stirrer for on-ice dialysis. PBS buffer was replaced every 4 h until no fluorescein was detected in the buffer. All operations were conducted under dark conditions. The concentration of the FITC-labeled Cry2Ab12 was re-determined, and the labeled protein was incorporated into an artificial aphid diet using a membrane vesicle device for *M. persicae* feeding. The control group received artificial diet only. The artificial diet was formulated based on various amino acids (e.g., Ala, Arg, Asn) at different ratios, supplemented with vitamins (e.g., ascorbic acid, calcium pantothenate, nicotinamide) and inorganic salts (e.g., NaCl, KH_2_PO_4_, FeCl_3_) [46]. After 40 min of feeding, Cry2Ab12 localization in *M. persicae* was observed using a laser scanning confocal microscope.

Bioassay of Cry2Ab12 toxin against *M. persicae*. First-instar nymphs and adult *M. persicae* were individually transferred into disposable transparent Petri dishes at a density of 25 aphids per dish, with six dishes prepared for each developmental stage. For the 50 μg/mL treatment group, artificial aphid diet and Cry2Ab12 toxin were sequentially added to a sterile 15 mL EP tube, mixed thoroughly by vortexing, and then poured into a 25 mL syringe connected to a bacterial filter mounted on another sterile 15 mL EP tube to filter the mixture into the new tube. For the control group, carbonate buffer was added at an equal volume in place of the protein solution. The filtered protein–diet mixture and buffer–diet mixture were each added to the parafilm-covered end of the corresponding labeled bioassay rings at 2 mL per ring. A second piece of stretched parafilm was promptly positioned over the top to distribute the mixture uniformly between the two layers. The bioassay rings were then placed on a lab bench lined with paper towels, and the 25 aphids from each Petri dish were transferred into the corresponding rings. Once the aphid count was confirmed, the other end of each ring was sealed with parafilm, which was then punctured 20–30 times with a needle to create air vents. Finally, the prepared bioassay rings were incubated in an intelligent artificial climate chamber set at 23 ± 2 °C, 75% relative humidity (RH), with a 16:8 light:dark (L:D) photoperiod. Survival rates were assessed after 3 d.

Protein pull-down experiments. After collecting approximately 200 fourth-instar *M. persicae* nymphs, the specimens were thoroughly pulverized in a tissue grinder with 500 μL of PBS buffer. The resulting homogenate was centrifuged (12,000 rpm, 4 °C, 10 min), and the soluble material from the middle layer was then subjected to pull-down experiments as described by Shu et al. [47]. In brief, 600 mg of Sepharose™ 4B beads (GE Healthcare, Beijng, China) were resuspended in 1 mmol/L HCl and pre-equilibrated with coupling buffer (0.1 M NaHCO_3_, 0.5 M NaCl, pH 8.3). A 1 mL aliquot of Cry2Ab12 toxin (0.5 mg/mL dissolved in 1 mmol/L HCl) was immediately combined with the beads, and the toxin-bead mixture was gently shaken at 4 °C for 12 h. Subsequently, 50 μL of *M. persicae* homogenate was added to 25 μL of the toxin-conjugated matrix and incubated for 1 h at 4 °C. Proteins interacting with Cry2Ab12 were then separated by 12% SDS-PAGE and visualized using a Fast Silver Stain Kit (Beyotime, Haimen, China). The entire experiment was conducted in triplicate. After silver staining, the unique protein bands were excised and analyzed using liquid chromatography-tandem mass spectrometry (LC-MS/MS) at the Huada Protein Research Center (HPRC). The LC-MS/MS spectra were then searched against the uni-aphidoidea_33385 database (52,503 entries; 17,662,111 residues) via the MASCOT search engine (Matrix Science, London, UK). Search parameters were configured as follows: search type, MS/MS Ion Search; enzyme, trypsin; fixed modification, carbamidomethylation of cysteine (C); Variable modifications, pyroglutamate formation from N-terminal glutamine (Gln → pyro-Glu) and methionine oxidation (M); monoisotopic mass values; no restriction on protein molecular weight; peptide mass tolerance set to ±15 ppm; fragment mass tolerance set to ±20 mmu; maximum allowable missed cleavages, 2; Instrument type left at default; total number of queries, 13,131.

Homology modeling and molecular docking. SWISS-MODEL (http://swissmodel.expasy.org) (accessed on 21 November 2021) [48] was used to predict the three-dimensional (3D) structures of both Cry2Ab12 toxin and OLA1. The MolProbity server (http://molprobity.biochem.duke.edu/) (accessed on 4 March 2022) [49] was then employed to evaluate model quality by submitting the PDB files. Finally, Z-dock (Discovery Studio 2018) was utilized to perform docking between Cry2Ab12 toxin and OLA1, with all parameters left at their default values [50].

RT-qPCR analysis. Real-time quantitative PCR (RT-qPCR) on a CFX Connect Real-Time System (Bio-Rad, Singapore) was employed to assess the expression of *M. persicae ola1* in response to Cry2Ab12 toxin. Nymph and adult stages of *M. persicae* were independently treated with Cry2Ab12 toxin at concentrations of 0, 25, and 50 μg/mL using a membrane capsule method. Following a 24 h incubation, total RNA was isolated with the RaPure Universal RNA Plus Kit (Magen Biotechnology, Guangzhou, China) and then reverse-transcribed into cDNA using the TransScript^®^ All-in-One First-Strand cDNA Synthesis SuperMix (TransGen Biotech, Beijing, China) in accordance with the manufacturer’s instructions. We designed primers using Primer 7.0 software and had them synthesized by TSINGKE (TSINGKE, Beijing, China) (Table 3). RT-qPCR reactions were run with the SYBR Green Pro Taq HS qPCR Kit (Accurate Biotechnology, Changsha, China) according to the following protocol: initial denaturation at 95 °C for 2 min, then 40 cycles at 95 °C for 5 s and 60 °C for 30 s. Relative quantification was determined by the 2^−ΔΔCt^ method [51]. Three technical and three biological replicates were performed per treatment.

Biosensor-binding kinetics. The Octet RED96e system (Pall ForteBio Corp., Menlo Park, CA, USA) [52,53] was employed to measure the binding kinetics between OLA1 and the Cry2Ab12 toxin. The researchers first loaded OLA1 (as the coupling sample) onto Aminopropylsilane (APS) biosensors for 500 s. Cry2Ab12 binding to the captured OLA1 was then assessed using a 500 s association phase and a 500 s dissociation phase. PBST (PBS, pH 7.4, 0.05% Tween-20) supplemented with 5% DMSO (*v*/*v*) served as the assay buffer. The Octet System Data Analysis Software 9.0 was used to calculate the dissociation equilibrium constant (KD) [54,55,56].

Effects of Cry2Ab12 toxin on OLA1 Activity. OLA1 activity was determined using a malachite green assay, with a standard curve established using KH_2_PO_4_ as the standard [57]. A series of dilutions was performed on KH_2_PO_4_, producing final concentrations of 100, 50, 25, 12.5, 6.25, and 3.12 μmol/L. Each dilution was mixed with a pre-incubated (≥20 min) and filtered (0.45 μm) mixture of the OLA1 enzyme activity reaction solution and 0.045% malachite green (1:3, *v*/*v*). Each reaction mixture was incubated for 15 min at ambient temperature. Subsequently, the OD_620_ absorbance was quantified using a spectrophotometer, with each reaction measured in triplicate. The resulting standard curve yielded the linear equation y = 0.00268x + 0.05344 (R^2^ = 0.99961). OLA1 harbors a GTPase active pocket capable of hydrolyzing GTP into GDP and inorganic phosphate, and its enzyme activity can be quantified via the malachite green assay. We monitored substrate hydrolysis by measuring absorbance changes at 37 °C. A UV-1800 spectrophotometer (Mapada, Shanghai, China) and a SPECORD 40 UV-Vis spectrophotometer (Analytik Jena AG, Jena, Germany), each equipped with a thermostatically controlled cuvette holder, were used for this purpose. Absorbance was measured at 620 nm. The reaction system (total volume 50 μL) consisted of 0.5 μmol/L OLA1, 500 μmol/L GTP, 25 mmol/L Hepes-KOH, and 100 mmol/L KCl, with Cry2Ab12 added as the test component. After adding 800 μL of the pre-prepared malachite green–OLA1 reaction mixture, incubation of the reaction was carried out at 37 °C for 15 min, and the process was halted by adding 100 μL of 34% sodium citrate. GTPase activity was calculated by referencing the KH_2_PO_4_ standard curve. All measurements were performed at least in triplicate.

Enzyme-linked immunosorbent assay (ELISA) Analysis. To examine the binding interactions between recombinant Cry2Ab12, HSP60, and OLA1, a modified microplate assay was performed according to a published protocol [58]. Briefly, each well of 96-well plates received 0.8 μg of recombinant Cry2Ab12 or HSP60 for overnight coating at 4 °C. The plates were then rinsed three times with PBST and subsequently blocked with 5% skim milk. Recombinant Cry2Ab12 and OLA1 were each conjugated to NHS-biotin (Thermo Fisher Scientific, Shanghai, China) and then purified using dialysis membranes with molecular weight cutoffs of 30 kDa and 10 kDa, respectively, as per the manufacturer’s protocols [59]. The pre-coated ELISA plates were then incubated for 2 h at 37 °C with serially diluted concentrations of biotinylated Cry2Ab12 or OLA1. Unbound section was removed by performing three washes with PBS (pH 7.4) and three additional washes with PBST. Detection of bound proteins was accomplished with a primary antibody (1:3000) (Proteintech Group, Chicago, IL, USA), followed by a secondary antibody conjugated to HRP (1:3000) (Proteintech Group, Chicago, IL, USA). Incubations with each antibody were conducted at 37 °C for 2 h. The chromogenic reagent kit EL-TMB P0209 (Beyotime Biotech, Haimen, China) was used for color development, and the reaction was terminated by the addition of 50 μL of 2 M H_2_SO_4_. Measurement of absorbance was performed at 450 nm using a ReadMax 1900 Multiskan microplate spectrophotometer (Flash Spectrum Biological Technology, Shanghai, China). Each treatment was performed in triplicate.

## Figures and Tables

**Table 1:** LC-MS/MS identification of Cry2Ab12 toxin-binding proteins in *M. persicae*.

Sample	Accession Number	Score	Protein Description
1	tr|D7RA98|D7RA98_ACYPI	373	Tubulin beta chain OS = *Acyrthosiphon pisum*
2	tr|C4WVW3|C4WVW3_ACYPI	289	elongation factor 1-gamma OS = *Acyrthosiphon pisum*
3	tr|C4WSE9|C4WSE9_ACYPI	217	Obg-like ATPase 1 OS = *Acyrthosiphon pisum*
4	tr|J9K1S3|J9K1S3_ACYPI	198	Elongation factor 1-alpha OS = *Acyrthosiphon pisum*
5	tr|J9K0B0|J9K0B0_ACYPI	86	26S protease regulatory OS = *Acyrthosiphon pisum*
6	tr|J9K663|J9K663_ACYPI	22	H/ACA ribonucleoprotein OS = *Acyrthosiphon pisum*

**Table 2:** Summary of Octet binding kinetic constants of Cry2Ab12 toxin to OLA1.

Sensor Type	Sample ID	Conc. (nM)	Response	K_D_ (M)	kon (1/Ms)	kdis (1/s)
SA	OLA1	1000	−0.001	7.83 × 10^−10^	1.00 × 10^−10^	7.83 × 10^−6^
SA	OLA1	500	−0.0039	7.83 × 10^−10^	1.00 × 10^−10^	7.83 × 10^−6^
SA	OLA1	250	0.0012	7.83 × 10^−10^	1.00 × 10^−10^	7.83 × 10^−6^
SA	OLA1	125	0.0031	7.83 × 10^−10^	1.00 × 10^−10^	7.83 × 10^−6^
SA	OLA1	62.5	0.0039	7.83 × 10^−10^	1.00 × 10^−10^	7.83 × 10^−6^

Note: the K_D_ value for protein–protein interaction is usually 10^−8^–10^−11^ M. If K_D_ value is >100 nM (10^−7^ M), the binding ability between the two proteins is relatively weak.

**Table 3:** RT-qPCR primers.

Primer Name	Sequence (5′ to 3′)
OLA1 (forward)	TGGAAGATGTTCAGGCTACAAGTGC
OLA1 (reverse)	TTGACTTCGTCATGTCCAGCAGTG

## Data Availability

The original contributions presented in this study are included in the article. Further inquiries can be directed to the corresponding authors.

## References

[1] Walters F.S., English L.H. (1995). Toxicity of *Bacillus thuringiensis* δ-endotoxins toward the potato aphid in an artificial diet bioassay. Entomol. Exp. Appl..

[2] Wei J.Z., Hale K., Carta L., Platzer E., Wong C., Fang S.C., Aroian R.V. (2003). *Bacillus thuringiensis* crystal proteins that target nematodes. Proc. Natl. Acad. Sci. USA.

[3] Jing X., Yuan Y., Wu Y., Wu D., Gong P., Gao M. (2018). Crystal structure of *Bacillus thuringiensis* Cry7Cal toxin active against *Locusta migratoria manilensis*. Protein Sci..

[4] Qaim M., Zilberman D. (2003). Yield Effects of Genetically Modified Crops in Developing Countries. Science.

[5] Soberón M., Rodriguez-Almazán C., Muñóz-Garay C., Pardo-López L., Porta H., Bravo A. (2012). *Bacillus thuringiensis* Cry and Cyt mutants useful to counter toxin action in specific environments and to overcome insect resistance in the field. Pestic. Biochem. Physiol..

[6] Sanahuja G., Banakar R., Twyman R.M., Capell T., Christou P. (2015). *Bacillus thuringiensis*: A century of research, development and commercial applications. Plant Biotechnol. J..

[7] Chougule N.P., Li H., Liu S., Linz L.B., Narva K.E., Meade T. (2013). Retargeting of the *Bacillus thuringiensis* toxin Cyt2Aa against hemipteran insect pests. Proc. Natl. Acad. Sci. USA.

[8] Ferr J., Escriche B., Bel Y., Rie J. (2002). Biochemistry and genetics of insect resistance to *Bacillus thuringiensis*. Ann. Rev. Entomol..

[9] Haonan Z., Wei Y., Jing Z., Lin J., Yihua Y., Shuwen W. (2011). Early Warning of Cotton Bollworm Resistance Associated with Intensive Planting of Bt Cotton in China. PLoS ONE.

[10] Gassmann A.J. (2012). Field-evolved resistance to Bt maize by western corn rootworm: Predictions from the laboratory and effects in the field. J. Invertebr. Pathol..

[11] Dhurua S., Gujar G.T. (2011). Field-evolved resistance to Bt toxin Cry1Ac in the pink bollworm, *Pectinophora gossypiella* (Saunders) (Lepidoptera: Gelechiidae), from india. Pest Manag. Sci..

[12] Palma L., Muñoz D., Berry C., Murillo J., Caballero P. (2014). *Bacillus thuringiensis* toxins: An overview of their biocidal activity. Toxins.

[13] Cristofoletti P.T., Ribeiro A.F., Deraison C., Yvan R., Terra W.R. (2003). Midgut adaptation and digestive enzyme distribution in a phloem feeding insect, the pea aphid *Acyrthosiphon pisum*. J. Insect Physiol..

[14] Deraison C., Darboux I., Duportets L., Gorojankina T., Jouanin L. (2004). Cloning and characterization of a gut-specific cathepsin L from the aphid *Aphis gossypii*. Insect Mol. Biol..

[15] Li H., Chougule N.P., Bonning B.C. (2011). Interaction of the *Bacillus thuringiensis* delta endotoxins Cry1Ac and Cry3Aa with the gut of the pea aphid, *Acyrthosiphon pisum* (Harris). J. Invertebr. Pathol..

[16] Torres-Quintero M.C., Arenas-Sosa I., Zuñiga-Navarrete F., Hernández-Velázquez V.M., Alvear-Garcia A., Peña-Chora G. (2022). Characterization of insecticidal Cry protein from *Bacillus thuringiensis* toxic to *Myzus persicae* (Sulzer). J. Invertebr. Pathol..

[17] Palma L., Muñoz D., Berry C., Murillo J., De Escudero I., Caballero P. (2014). Molecular and insecticidal characterization of a novel Cry-related protein from *Bacillus thuringiensis* toxic against *Myzus persicae*. Toxins.

[18] Zhao X.D., Zhang B.W., Fu L.J., Li Q.L., Lin Y., Yu X.Q. (2020). Possible insecticidal mechanism of Cry41-related toxin against *Myzus persicae* by enhancing cathepsin B activity. J. Agric. Food Chem..

[19] Russo A.A., Jeffrey P.D., Patten A.K., Massagué J., Pavletich N.P. (1996). Crystal structure of the p27Kip1 cyclin-dependent-kinase inibitor bound to the cyclin A-Cdk2 complex. Nature.

[20] Prince V.S.J., Valentina R., Chen H., Zhang J.W., Shi Z.Z. (2014). Regulation of cell-matrix adhesion by OLA1, the Obg-like ATPase 1. Biochem. Biophys. Res. Commun..

[21] Chen H., Song R., Wang G., Ding Z., Yang C., Zhang J. (2015). OLA1 regulates protein synthesis and integrated stress response by inhibiting eIF2 ternary complex formation. Sci. Rep..

[22] Ding Z., Liu Y., Rubio V., He J., Shi Z.Z. (2016). OLA1, a Translational Regulator of p21, Maintains Optimal Cell Proliferation Necessary for Developmental Progression. Mol. Cell. Biol..

[23] Taulés M., Rodríguez-Vilarrupla A., Rius E., Estanyol J.M., Casanovas O., Sacks D.B., Pérez-Payá E., Bachs O., Agell N. (1999). Calmodulin Binds to p21Cip1 and Is Involved in the Regulation of Its Nuclear Localization. J. Biol. Chem..

[24] Fuxreiter M., Tompa P. (2012). Fuzzy Complexes: A More Stochastic View of Protein Function. Adv. Exp. Med. Biol..

[25] Agell N., Jaumot M., Rodríguez-Vilarrupla A., Brun S., Abella N., Canela N., Estanyol J.M., Bachs O. (2006). The diverging roles of calmodulin and PKC in the regulation of p21 intracellular localization. Cell Cycle.

[26] Ashcroft M., Taya Y., Vousden K.H. (2000). Stress signals utilize multiple pathways to stabilize p53. Mol. Cell Biol..

[27] Huang S., Zhang C., Sun C., Hou Y., Zhang Y., Tam N.L., Wang Z., Yu J., Huang B., Zhuang H. (2020). Obg-like ATPase 1 (OLA1) overexpression predicts poor prognosis and promotes tumor progression by regulating P21/CDK2 in hepatocellular carcinoma. Aging.

[28] Mao R.F., Rubio V., Chen H., Bai L., Mansour O.C., Shi Z.Z. (2013). OLA1 protects cells in heat shock by stabilizing HSP70. Cell Death Dis..

[29] Schultz A., Olorundami O.A., Teng R.J., Jarzembowski J., Shi Z.Z., Kumer K.P., Konduri G.G., Afolayan A.J. (2019). Decreased OLA1 (Obg-Like ATPase-1) expression drives ubiquitin-proteasome pathways to downregulate mitochondrial SOD2 (superoxide dismutase) in persistent pulmonary hypertension of the newborn. Hypertension.

[30] Guerrero C., Tagwerker C., Kaiser P., Huang L. (2006). An integrated mass spectrometry-based proteomic approach: Quantitative analysis of tandem affinity-purified in vivo cross-linked protein complexes (QTAX) to decipher the 26S proteasome-interacting network. Mol. Cell. Proteom..

[31] Guerrero C., Milenkovic T., Przulj N., Kaiser P., Huang L. (2008). Characterization of the proteasome interaction network using a QTAX-based tag-team strategy and protein interaction network analysis. Proc. Natl. Acad. Sci. USA.

[32] Wang X., Huang L. (2008). Identifying dynamic interactors of protein complexes by quantitative mass spectrometry. Mol. Cell. Proteom..

[33] Leipe D.D., Wolf Y.I., Koonin E.V., Aravind L. (2002). Classification and evolution of P-loop GTPases and related ATPases. J. Mol. Biol..

[34] Maagd D.R.A., Bakker P.L., Masson L., Adang M.J., Sangadala S., Stiekema W., Bosch D. (1999). Domain III of the *Bacillus thuringiensis* delta-endotoxin Cry1Ac is involved in binding to *Manduca sexta* brush border membranes and to its purified aminopeptidase N. Mol. Microbiol..

[35] Vetter I.R., Wittinghofer A. (2001). The Guanine Nucleotide-Binding Switch in Three Dimensions. Science.

[36] Minakuchi C., Namiki T., Yoshiyama M., Shinoda T. (2010). RNAi-mediated knockdown of juvenile hormone acid O-methyltransferase gene causes precocious metamorphosis in the red flour beetle *Tribolium castaneum*. FEBS J..

[37] Kozlova T., Perezgasga L., Reynaud E., Zurita M. (1997). The *Drosophila melanogaster* homologue of the hsp60 gene is encoded by the essential locus l(1)10Ac and is differentially expressed during fly development. Dev. Genes Evol..

[38] Jin L., Zhao X.D., Lu J.W., Zhang B.W., Lei Y., Lin Y. (2023). Protection of HSP60 in *Myzus persicae* Treated with Cry7Ab4 Toxin. ACS Agric. Sci. Technol..

[39] Bravo A., Likitvivatanavong S., Gill S.S., Soberon M. (2011). *Bacillus thuringiensis*: A story of a successful bioinsecticide. Insect Biochem. Mol. Biol..

[40] Wenk M., Ba Q., Erichsen V., Macinnes K., Wiese H., Warscheid B. (2012). A universally conserved ATPase regulates the oxidative stress response in *Escherichia coli*. J. Biol. Chem..

[41] Meier U.T. (2005). The many facets of H/ACA ribonucleoprotein. Chromosoma.

[42] Dubiel W., Ferrell K., Rechsteiner M. (1995). Subunits of the regulatory complex of the 26S protease. Mol. Biol. Rep..

[43] Collins A.G., Goldberg A.L. (2017). The Logic of the 26S Proteasome. Cell.

[44] Yang Z., Zhang L., Zhang Y., Zhang T., Feng Y., Lu X., Lan W., Wang J., Wu H., Cao C. (2011). Highly efficient production of soluble proteins from insoluble inclusion bodies by a two-stepdenaturing and refolding method. PLoS ONE.

[45] Linova M.Y., Risør M.W., Jørgensen S.E., Mansour Z., Kaya J., Sigurdarson J.J., Enghild J.J., Karring H. (2019). A novel approach for production of an active N-terminally truncated Ulp1 (SUMO protease 1) catalytic domain from *Escherichia coli* inclusion bodies. Protein Expr. Purif..

[46] Jin L., Zhang B.W., Aguila L.C.R., Lu J.W., Gao X.K., Luo J.Y., Cui J.J., Lin Y. (2025). Potential Mechanisms Underlying the Minimal Impact of Cry1Ab1 Protein on *Myzus persicae*. Int. J. Mol. Sci..

[47] Shu C., Tan S., Yin J., Soberon M., Bravo A., Liu C., Geng L., Song F., Li K., Zhang J. (2015). Assembling of *Holotrichia parallela* (dark black chafer) midgut tissue transcriptome and identification of midgut proteins that bind to Cry8Ea toxin from *Bacillus thuringiensis*. Appl. Microbiol. Biotechnol..

[48] Biasini M., Bienert S., Waterhouse A., Arnold K., Studer G., Schmidt T., Kiefer F., Cassarino T.G., Bertoni M., Bordoli L. (2014). SWISS-MODEL: Modelling protein tertiary and quaternary structure using evolutionary information. Nucleic Acids Res..

[49] Chen V.B., Arendall W.B., Headd J.J., Keedy D.A., Immormino R.M., Kapral G.J., Murray L.W., Richardson J.S., Richardson D.C. (2010). MolProbity: All-atom structure validation for macromolecular crystallography. Acta Crystallogr. Sect. D Biol. Crystallogr..

[50] Pierce B.G., Wiehe K., Hwang H., Kim B.-H., Vreven T., Weng Z. (2014). ZDOCK server: Interactive docking prediction of protein-protein complexes and symmetric multimers. Bioinformatics.

[51] Livak K.J., Schmittgen T.D. (2001). Analysis of Relative Gene Expression Data Using Real-Time Quantitative PCR and the 2^−ΔΔCT^ Method. Methods.

[52] Li Z., Munro K., Narouz M.R., Lau A., Hao H., Crudden C.M., Horton J.H. (2018). Self-Assembled N-Heterocyclic Carbene-Based Carboxymethylated Dextran Monolayers on Gold as a Tunable Platform for Designing Affinity-Capture Biosensor Surfaces. ACS Appl. Mater. Interfaces.

[53] Sharma S., Oot R.A., Wilkens S. (2018). MgATP hydrolysis destabilizes the interaction between subunit H and yeast V1-ATPase, highlighting H’s role in V-ATPase regulation by reversible disassembly. J. Biol. Chem..

[54] Gates Z.P., Vinogradov A.A., Quartararo A.J., Bandyopadhyay A., Choo Z.-N., Evans E.D., Halloran K.H., Mijalis A.J., Mong S.K., Simon M.D. (2018). Xenoprotein engineering via synthetic libraries. Proc. Natl. Acad. Sci. USA.

[55] Ma D., Wang Z., Merrikh C.N., Lang K.S., Lu P., Li X., Merrikh H., Rao Z., Xu W. (2018). Crystal structure of a membrane-bound O-acyltransferase. Nature.

[56] Callaway H.M., Welsch K., Weichert W., Allison A.B., Hafenstein S.L., Huang K., Iketani S., Parrish C.R. (2018). Complex and Dynamic Interactions between Parvovirus Capsids, Transferrin Receptors, and Antibodies Control Cell Infection and Host Range. J. Virol..

[57] Zhang X.X., Yan K., Zhang Y.X., Li N.N., Ma C.Y., Li Z.F., Zhang Y.Q., Feng B.Y., Liu J., Sun Y.D. (2014). Structural insights into the function of a unique tandem GTPase EngA in bacterial ribosome assembly. Nucleic Acids Res..

[58] Batool K., Alam I., Jin L., Xu J., Wu C., Wang J., Huang E., Guan X., Yu X.-Q., Zhang L. (2019). CTLGA9 Interacts with ALP1 and APN Receptors to Modulate Cry11Aa Toxicity in *Aedes aegypti*. J. Agric. Food Chem..

[59] Zhang L., Zhao G., Hu X., Liu J., Li M., Batool K., Chen M., Wang J., Xu J., Huang T. (2017). Cry11Aa Interacts with the ATP-Binding Protein from *Culex quinquefasciatus* to Improve the Toxicity. J. Agric. Food Chem..

